# MiR-195 inhibits the ubiquitination and degradation of YY1 by Smurf2, and induces EMT and cell permeability of retinal pigment epithelial cells

**DOI:** 10.1038/s41419-021-03956-6

**Published:** 2021-07-15

**Authors:** Shu-Hua Fu, Mei-Chen Lai, Yun-Yao Zheng, Ya-Wen Sun, Jing-Jing Qiu, Fu Gui, Qian Zhang, Fei Liu

**Affiliations:** Department of Ophthalmology, The Second Affiliated Hospital of Nanchang University, Nanchang, 330006 Jiangxi Province P. R. China

**Keywords:** Mechanisms of disease, Diabetes

## Abstract

The dysregulated microRNAs (miRNAs) are involved in diabetic retinopathy progression. Epithelial mesenchymal transition (EMT) and cell permeability are important events in diabetic retinopathy. However, the function and mechanism of miR-195 in EMT and cell permeability in diabetic retinopathy remain largely unclear. Diabetic retinopathy models were established using streptozotocin (STZ)-induced diabetic mice and high glucose (HG)-stimulated ARPE-19 cells. Retina injury was investigated by hematoxylin–eosin (HE) staining. EMT and cell permeability were analyzed by western blotting, immunofluorescence, wound healing, and FITC-dextran assays. MiR-195 expression was detected via qRT-PCR. YY1, VEGFA, Snail1, and Smurf2 levels were detected via western blotting. The interaction relationship was analyzed via ChIP, Co-IP, or dual-luciferase reporter assay. The retina injury, EMT, and cell permeability were induced in STZ-induced diabetic mice. HG induced EMT and cell permeability in ARPE-19 cells. MiR-195, YY1, VEGFA, and Snail1 levels were enhanced, but Smurf2 abundance was reduced in STZ-induced diabetic mice and HG-stimulated ARPE-19 cells. VEGFA knockdown decreased Snail1 expression and attenuated HG-induced EMT and cell permeability. YY1 silence reduced VEGFA and Snail1 expression, and mitigated HG-induced EMT and cell permeability. YY1 could bind with VEGFA and Snail1, and it was degraded via Smurf2-mediated ubiquitination. MiR-195 knockdown upregulated Smurf2 to decrease YY1 expression and inhibited HG-induced EMT and cell permeability. MiR-195 targeted Smurf2, increased expression of YY1, VEGFA, and Snail1, and promoted HG-induced EMT and cell permeability. MiR-195 promotes EMT and cell permeability of HG-stimulated ARPE-19 cells by increasing VEGFA/Snail1 via inhibiting the Smurf2-mediated ubiquitination of YY1.

## Introduction

Diabetic retinopathy is a common clinical disease occurring in ~35% diabetic patients, with hyperglycemia as a main risk, which can induce blindness or severe vision impairment [1]. The retinal pigment epithelium is located between the choriocapillaris and the outer segment of photoreceptors, which is responsible for diabetic retinopathy development [2]. Epithelial mesenchymal transition (EMT) is involved in the formation of fibrous epiretinal membranes in diabetic retinopathy, which is induced by high glucose (HG) [3]. Moreover, the permeability by disturbing the blood-retinal barrier cell junctions is also an important feature in diabetic retinopathy [4]. Hence, exploring the mechanism of EMT and permeability of retinal pigment epithelial cells may contribute to find a promising strategy for diabetic retinopathy therapy.

Gene-based therapy may open new opportunity for diabetic retinopathy treatment [5]. Snail1 is an important transcriptional suppressor of E-cadherin, which contributes to EMT in retinal pigment epithelium cells [6, 7]. Furthermore, Snail1 can increase cell permeability by regulating tight junction proteins [8]. Vascular endothelial growth factor A (VEGFA) is a key factor promoting retinal vascular permeability in retinal vascular diseases [9] and the VEGFA/VEGFR2 signaling is associated with HG-induced EMT and permeability of retinal pigment epithelial cells [10]. Moreover, VEGFA stimulates Snail1 expression to drive EMT in cancer cells [11, 12]. Yin Yang 1 (YY1) is an important transcription factor ubiquitously expressed in mammalian cells, which can modulate transcription activation or repression [13]. Previous reports report that YY1 can bind with the promoter of VEGFA and Snail1 [14, 15]. However, whether YY1 can target VEGFA/Snail1 axis to participate in EMT and permeability of retinal pigment epithelial cells is unknown.

MicroRNAs (miRNAs) are short noncoding RNAs that can repress protein translation or degrade mRNA by binding with the 3′-untranslated region (UTR) of targets, which have important roles in diabetic retinopathy development [16]. In addition, the dysregulated miRNAs are associated with EMT process in retinal pigment epithelial cells [17]. MiR-195 is increased in HG-treated cells and contributes to retinal endothelial cell injury in diabetic retinopathy [18, 19]. The Smad ubiquitin regulatory factor 2 (Smurf2) is a common ubiquitinase that regulates protein homeostasis [20]. Moreover, Smurf2 can induce the ubiquitination and degradation of YY1 [21]. In addition, Smurf2 can be targeted and inhibited via miR-195 in lung cancer cells [22]. Hence, we hypothesized that miR-195 might regulate YY1 indirectly by targeting Smurf2 to modulate VEGFA and Snail1 expression, thus participating in EMT and permeability of retinal pigment epithelial cells. Yet, no study has reported this network in diabetic retinopathy.

In this study, we established the streptozotocin (STZ)-induced diabetic murine model and HG-stimulated retinal pigment epithelial cellular model. We mainly aimed to investigate the function of VEGFA/Snail1 axis, YY1, and miR-195 on EMT and permeability of retinal pigment epithelial cells under HG condition, and to confirm the mechanism was associated with the miR-195/Smurf2/YY1/VEGFA/Snail1 axis.

## Materials and methods

### Bioinformatics analysis

The binding of YY1 and the promoter of VEGFA, Snail1, or Vimintin was predicted via JASPAR (http://jaspar.genereg.net/). The target sites of miR-195 on Smurf2 were predicted by StarBase (http://starbase.sysu.edu.cn/index.php).

### Animal model

The 10-week-old male C57BL/6J mice were purchased from Charles river (Beijing, China) and were divided into control or model group (*n* = 7 per group). The diabetes was induced via intraperitoneal injection of 55 mg/kg STZ for 5 days and was maintained for 6 months. The mice with three consecutive measurements of blood glucose > 275 mg/dL were regarded as the diabetic model group. The age-matched normal male mice were used as controls. After 6 months of diabetes, mice were killed via inhalation anesthesia of 5% isoflurane. The retinal tissues were collected for retinal histopathology via hematoxylin–eosin (HE) staining and were used for RNA or protein detection. Animal procedures were approved via the Institutional Animal Care and Use Committee of the Second Affiliated Hospital of Nanchang University and were processed in accordance with the guideline of Animal Care and Use Committee of the National Institutes of Health.

### HE staining

The retinas were fixed with 4% paraformaldehyde (Beyotime, Shanghai, China), dehydrated and embedded in paraffin, followed via cutting in 5 μm sections. The sections were incubated in HE (Beyotime) for 5 min and imaged under a microscope (Olympus, Tokyo, Japan).

### Cell culture and HG treatment

Adult Retinal Pigment Epithelial cell line-19 (ARPE-19) cells and HEK293T cell lines were purchased from Procell (Wuhan, China), and were cultured in Dulbecco’s modified Eagle’s medium (DMEM) (containing 5 mM glucose) (Thermo Fisher, Waltham, MA, USA) plus 10% fetal bovine serum (HyClone, Logan, UT, USA) and 1% penicillin/streptomycin (Thermo Fisher) in 5% CO_2_ at 37 °C.

To induce diabetic retinopathy model, ARPE-19 cells were incubated with higher doses (15, 25, or 35 mM) of glucose for 0, 24, 48, and 72 h. The 35 mM of glucose-treated cells were regarded as the HG group. Cells were observed under a microscope.

### Cell transfection

YY1 (Myc-tagged YY1) overexpression vector was constructed via inserting the sequence of YY1 in pcDNA3.1 vector (Thermo Fisher), with the empty vector (EV) as the negative control (NC). Short hairpin RNA (shRNA) for VEGFA (shVEGFA), shRNA for YY1 (shYY1), shRNA NC (shNC), small interfering RNA for Smurf2 (siSmurf2), Scramble, miR-195 inhibitor, inhibitor NC, miR-195 mimics, and mimics NC were generated via GenePharma (Shanghai, China) and the oligonucleotides sequences were shown in Table 1. ARPE-19 cells were transfected with 20 nM of oligonucleotides or 600 ng vectors using Lipofectamine 3000 (Thermo Fisher) for 24 h. MG132 was purchased from Sigma.

**Table 1:** The sequences for oligos in this study.

Name	Sequence (5′–3′)
shVEGFA	AGAACUAGUGGUUUCAAUGGU
shYY1	UUUUGAAACGAGAUUACAGAG
shNC	AAGACAUUGUGUGUCCGCCTT
siSmurf2	AUUGAUGUUGCUAAGAUCCCU
Scramble	UUCUCCGAACGUGUCACGUTT
miR-195 mimics	UAGCAGCACAGAAAUAUUGGC
Mimics NC	CGAUCGCAUCAGCAUCGAUUGC
miR-195 inhibitor	GCCAAUAUUUCUGUGCUGCUA
Inhibitor NC	CUAACGCAUGCACAGUCGUACG

### Quantitative reverse-transcription PCR

The RNA was isolated using miRNA isolation kit (Thermo Fisher) according to the instructions and 500 ng RNA was reversely transcribed to cDNA with TaqMan miRNA reverse transcription kit (Thermo Fisher). The cDNA was mixed with SYBR (Takara, Otsu, Japan) and specific primers (Sangon, Shanghai, China). The mixture was used for quantitative reverse-transcription PCR on ABI Prism7500 Fast Real-Time PCR system (Applied Biosystems, Foster City, CA, USA). The primer pairs were displayed in Table 2. Relative miR-195 expression was calculated by 2^−ΔΔCt^ method with U6 as a reference.

**Table 2:** The primer sequences for qRT-PCR in this study.

Gene	Sequence	
	Forward (5′–3′)	Reverse (5′–3′)
hsa-miR-195	GCCGAGTAGCAGCACAGAAA	CAGTGCGTGTCGTGGAGT
mmu-miR-195	GCCGAGTAGCAGCACAGAAA	CAGTGCGTGTCGTGGAGT
hsa-U6	CTCGCTTCGGCAGCACA	AACGCTTCACGAATTTGCGT
mmu-U6	GCAAATTCGTGAAGCGTTCC	GCATAGACCTGAATGGCGGTA

### Wound-healing analysis

For cell migration assay, 1 × 10^5^ ARPE-19 cells were cultured in the six-well plates until 90% confluence. The straight wound was made through a pipette tip. Next, cells were incubated for 24 h. The wound was imaged by a microscope at 0 and 24 h. The migratory ability was investigated by the wound-healing ratio.

### Transwell migration assay

Transwell chamber with 8 μm pore size (Corning Incorporated, USA) was adopted to estimate the cell migration of ARPE-19 cells. Briefly, 500 μl DMEM containing 2 × 10^5^ treated ARPE-19 cells were added into the upper chamber, whereas 500 μl serum including (10%) DMEM was added into the lower one. After 24 h of culture, cells remained in the upper surface were discarded and those cells on the undersurface were fixed and stained with 5% crystal violet followed by number counting manually.

### Western blotting

Retinal tissues or ARPE-19 cells were lysed in radio-immunoprecipitation assay buffer (RIPA buffer, Beyotime, Shanghai, China). Protein samples were collected from lysates and were quantified through a bicinchoninic acid kit (Beyotime). Sodium dodecyl sulfate-polyacrylamide gel electrophoresis was conducted to separate the protein samples (20 μg) and then membrane transfer was performed using polyvinylidene difluoride membranes (Bio-Rad, Hercules, CA, USA). The nonspecific sites were blocked using 3% bovine serum albumin (BSA; Solarbio, Beijing, China). After the blockage for 1 h, membranes were incubated with primary antibodies overnight and secondary antibody for 2 h. The antibodies included anti-Smurf2 (ab94483, 1 : 500 dilution, Abcam, Cambridge, UK), anti-YY1 (ab227269, 1 : 1000 dilution, Abcam), anti-VEGFA (ab52917, 1 : 5000 dilution, Abcam), anti-Snail1 (#14-9859-80, 1 : 1000 dilution, Thermo Fisher), anti-Occludin (ab216327, 1 : 1000 dilution, Abcam), anti-E-cadherin (ab231303, 1 : 1000 dilution, Abcam), anti-N-cadherin (ab18203, 1 : 500 dilution, Abcam), anti-Vimentin (ab137321, 1 : 1000 dilution, Abcam), anti-Flag(ab236777, 1 : 1000 dilution, Abcam), anti-Myc(ab9106, 1 : 500 dilution, Abcam), anti-HA(ab18181, 1 : 1000 dilution, Abcam), anti-glyceraldehyde 3-phosphate dehydrogenase (GAPDH) (ab37168, 1 : 3000 dilution, Abcam), and horseradish peroxidase-conjugated IgG (ab205718/ab6789, 1 : 8000 dilution, Abcam). The blots were developed via enhanced chemiluminescence reagent (Beyotime) and were analyzed via Quantity One software (Bio-Rad), with GAPDH as a normalized control.

### Immunofluorescence

ARPE-19 cells were fixed with 4% paraformaldehyde and treated via 0.3% Triton X-100 (Solarbio), followed via blocked in 3% BSA for 30 min. Next, cells were incubated with the primary antibodies [anti-VEGFA (ab52917, 1 : 300 dilution, Abcam), anti-Snail1 (#14-9859-80, 1 : 100 dilution, Thermo Fisher), anti-Occludin (ab216327, 1 : 100 dilution, Abcam), anti-Vimentin (ab137321, 1 : 100 dilution, Abcam), or anti-E-cadherin (ab40772, 1 : 500 dilution, Abcam)] for 4 h and then incubated with IgG conjugated by Alexa Fluor® 647 (ab150079, 1 : 1000 dilution, Abcam) for 1 h. The nuclei were stained by DAPI (Solarbio). Cells were observed through a fluorescence microscope (Olympus).

### Fluorescein isothiocyanate-dextran assay

ARPE-19 cells (1 × 10^4^) were added into Transwell chamber (Corning, Corning, NY, USA). After cultured for 48 h, the medium was removed and 0.01% fluorescein isothiocyanate (FITC)-dextran (Sigma-Aldrich, St. Louis, MO, USA) was added in the upper chamber. After 60 min, the medium in the lower chamber was collected and the fluorescence intensity was detected with a fluorescence microplate reader (Molecular Devices, Sunnyvale, CA, USA) and normalized to the control group.

### Chromatin immunoprecipitation assay

To analyze the binding potential of YY1 and Snail1, Vimentin, or VEGFA promoter, Chromatin immunoprecipitation (ChIP) analysis were conducted with an Agarose ChIP kit (Thermo Fisher) according to the protocols. In brief, the crosslinking of 1 × 10^7^ ARPE-19 cells were processed by 1% formaldehyde (Beyotime) for 10 min, which was terminated via 0.125 M Glycine (Thermo Fisher). After the cell lysis, the chromatin was sheared via sonication to induce DNA fragments of 200–1000 bp. The lysates were incubated with the antibodies for YY1 (ab227269, 1 : 100 dilution, Abcam) overnight and then interacted with the Protein A/G-agarose beads for 6 h. Next, the beads were eluted and crosslinking was reversed via incubation at 65 °C for 4 h. The purified DNA was used to detected Snail1, Vimentin, or VEGFA promoter level by PCR. The primer sequences for the promoter were shown as: Snail1 promoter (forward: 5′-CATCCCTGGAAGCTGCTC-3′; reverse: 5′-GGGGTGACTTCCCAGAGG-3′), Vimentin promoter (forward: 5′-AACTTAGGGGCGCTCTTGTC-3′; reverse: 5′-TCTGTCGAGGGACCTAACGG-3′), and VEGFA promoter (forward: 5′-GGCTCTCTGTACATGAAGCAACT-3′; reverse: 5′-CCTAGTGACTGCCGTCTGC-3′). The enrichment level of Snail1, Vimentin, or VEGFA promoter was expressed as a fold change with IgG as a normalized group.

### Co-immunoprecipitation assay

Co-immunoprecipitation (Co-IP) assay was performed using EZ-Magna RIP™ RNA-Binding Protein Immunoprecipitation Kit (Sigma-Aldrich). In brief, ARPE-19 cells (1 × 10^7^) were lysed and then incubated with antibodies for YY1 (ab227269, 1 : 100 dilution, Abcam) or IgG overnight. Then the complex was incubated with the Protein A/G-agarose beads for 4 h. The YY1 and Smurf2 protein levels were detected via western blotting.

### Dual-luciferase reporter analysis

The wild-type (WT) sequence of Snail1 or VEGFA promoter containing YY1-binding sites, or Smurf2 having miR-195 binding sites, was inserted in the pGL3 luciferase reporter vectors (Promega, Madison, WI, USA) to generate the WT-Snail1, WT-VEGFA, or Smurf2 WT luciferase report vector. The mutant (Mut) Mut-Snail1, Mut-VEGFA, or Smurf2 Mut luciferase report vector was constructed by changing the corresponding binding sites. To analyze the binding of YY1 on Snail1 or VEGFA promoter, ARPE-19 cells were co-transfected with WT-Snail1, Mut-Snail1, WT-VEGFA, or Mut-VEGFA, and YY1 overexpression vector or EV using Lipofectamine 3000. To detect the target of miR-195 on Smurf2, ARPE-19 cells were co-transfected with Smurf2 WT or Smurf2 Mut, and miR-195 mimics or mimics NC using Lipofectamine 3000. After 24 h, luciferase activity was measured by dual-luciferase analysis kit (Promega).

### YY1 ubiquitination assay

HEK293T cells were transiently co-transfected with Myc-Smurf2, Flag-YY1, and HA-ubiquitin. After 48 h of transfection, cells were collected and lysed in RIPA buffer (Beyotime), and the cell lysates were subjected for IP using anti-Flag, which was followed by western blotting using anti-HA or anti-Flag antibody.

### Statistical analysis

The experiments were reperformed three times. The results were shown as mean ± SD and analyzed via GraphPad Prism 8 (GraphPad, Inc., La Jolla, CA, USA). The difference was compared by Student’s *t*-test for two groups, or was compared via analysis of variance followed via Tukey’s post hoc test for multiple groups. It was significant at *P* < 0.05.

## Results

### MiR-195, YY1, VEGFA, and Snail1 levels are increased, whereas Smurf2 expression is decreased in STZ-induced diabetic mice and HG-stimulated ARPE-19 cells

**Fig. 1:**
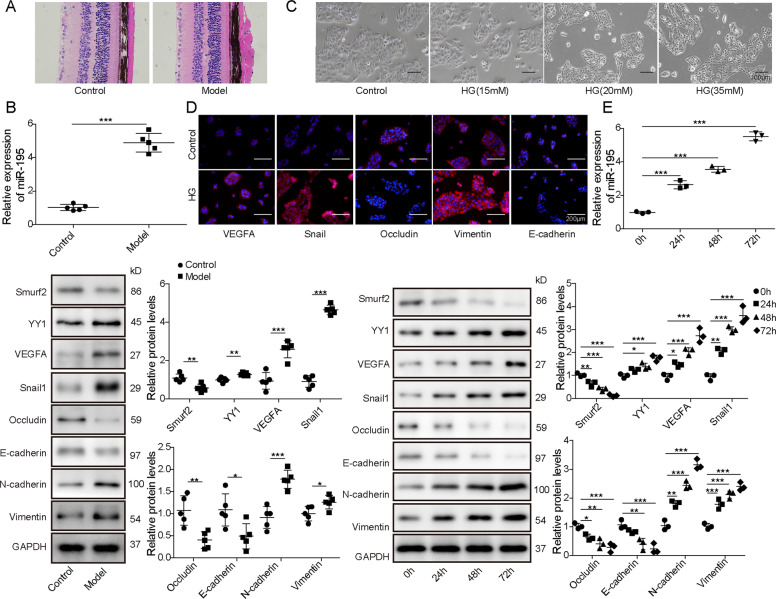
The expression of miR-195, YY1, VEGFA, Snail1, and Smurf2 in STZ-induced diabetic mice and HG-stimulated ARPE-19 cells. **A** The pathology of retinas from control or model group was analyzed by HE staining. **B** MiR-195, Smurf2, YY1, VEGFA, Snail1, Occludin, E-cadherin, N-cadherin, and Vimentin levels were detected via qRT-PCR or western blotting in the retinas from control or model group. **C** Cell morphology was observed under microscope in ARPE-19 cells after stimulation of different doses of glucose. Magnification: ×100. **D** VEGFA, Snail1, Occludin, E-cadherin, and Vimentin levels were examined by immunofluorescence in ARPE-19 cells in control or HG group. Magnification: ×200. **E** MiR-195, Smurf2, YY1, VEGFA, Snail1, Occludin, E-cadherin, N-cadherin, and Vimentin levels were examined by qRT-PCR or western blotting in ARPE-19 cells after 0, 24, 48, and 72 h of HG exposure. For each analysis, three technical replicates were performed and three biological independently performed replicates are included, *p < 0.05, **p < 0.01, ***p < 0.001.

To explore whether miR-195, YY1, VEGFA, Snail1, and Smurf2 were involved in diabetic retinopathy progression, their expression levels were detected in STZ-induced diabetic mice and HG-stimulated ARPE-19 cells. First, STZ-induced diabetic model was established. The morphological assay of HE-stained retinal tissues showed distortion of outer retinal layer in model group and normal retinal morphology was shown in control group (Fig. 1A). Moreover, miR-195, YY1, VEGFA, Snail1, Smurf2, and proteins associated with EMT and cell permeability were detected in retinal tissues. Results showed miR-195, YY1, VEGFA, Snail1, N-cadherin, and Vimentin levels were evidently enhanced, but Smurf2, Occludin, and E-cadherin abundances were markedly reduced in model group compared with control group (Fig. 1B). Next, ARPE-19 cells were treated via different doses of glucose for 48 h. Compared with control (5 mM) group, higher doses of glucose exposure increased the intercellular space in a dose-dependent pattern and resulted in fusiform or polygonal morphology (Fig. 1C). The highest dose (35 mM) of glucose was selected for further experiments and was set on HG group. Furthermore, immunofluorescence assay displayed VEGFA, Snail1, and Vimentin levels were evidently increased, but Occludin and E-cadherin levels were clearly decreased in the HG group compared with that in the control group (Fig. 1D). In addition, miR-195, YY1, VEGFA, Snail1, Smurf2, and proteins associated with EMT and cell permeability were measured in ARPE-19 cells after 0, 24, 48, and 72 h of HG exposure. As shown in Fig. 1E, with the HG exposure time prolonged, miR-195, YY1, VEGFA, Snail1, N-cadherin, and Vimentin were gradually increased, whereas Smurf2, Occcludin, and E-cadherin were gradually decreased in ARPE-19 cells. These results suggested miR-195, YY1, VEGFA, Snail1, and Smurf2 might be related to diabetic retinopathy progression.

### VEGFA silence downregulates Snail1 expression and represses HG-induced EMT and cell permeability of ARPE-19 cells

**Fig. 2:**
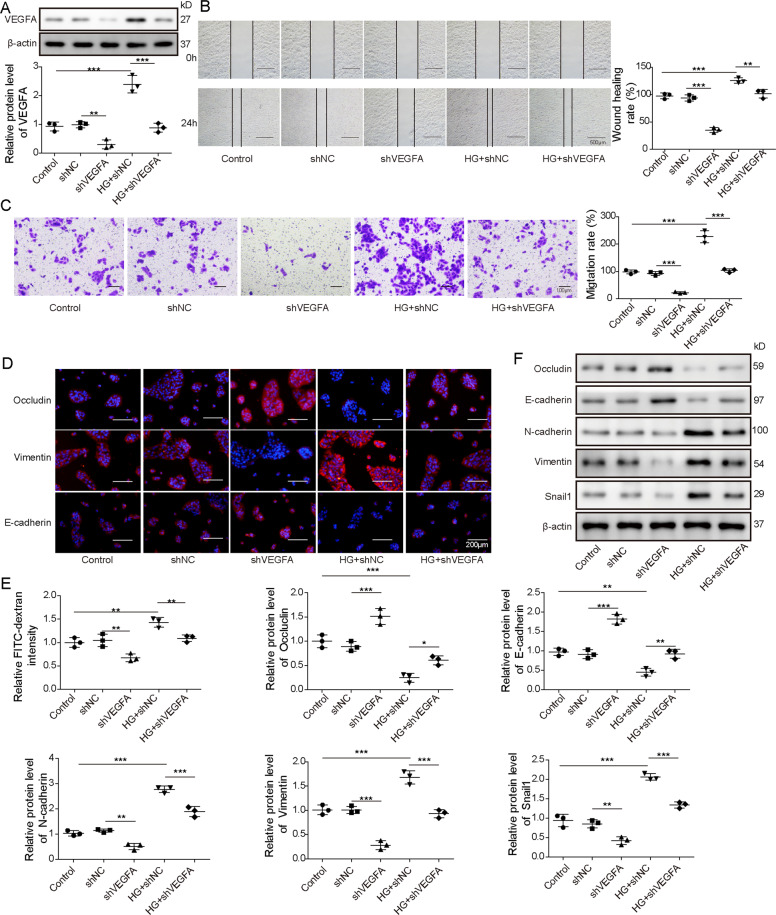
The suppressive effect of VEGFA silence on Snail1 expression and HG-induced EMT and cell permeability of ARPE-19 cells. ARPE-19 cells were transfected with shNC or shVEGFA and were then stimulated or not stimulated by HG. **A** VEGFA expression was measured by western blotting in the treated cells. **B**, **C** The migratory ability was detected via wound-healing analysis and transwell assay in the treated cells. **D** Occludin, E-cadherin, and Vimentin levels were examined by immunofluorescence in the treated cells. **E** Cell permeability was measured via FITC-dextran assay in the treated cells. **F** Snail1, Occludin, E-cadherin, N-cadherin, and Vimentin levels were detected via western blotting in the treated cells. For each analysis, three technical replicates were performed and three biological independently performed replicates are included, *p < 0.05, **p < 0.01, ***p < 0.001.

To study the function of VEGFA in HG-stimulated ARPE-19 cells, ARPE-19 cells were transfected with shNC or shVEGFA before HG stimulation. The transfection of shVEGFA effectively decreased VEGFA expression in ARPE-19 cells and this effect was reversed by HG stimulation (Fig. 2A). Moreover, wound-healing and transwell assays showed VEGFA knockdown evidently decreased ARPE-19 cell migration, and HG stimulation markedly promoted the migratory ability and abolished the effect of VEGFA silence (Fig. 2B, C). In addition, permeability-associated markers were examined via immunofluorescence assay. Results showed VEGFA knockdown led to obvious elevation of Occludin and E-cadherin, and reduction of Vimentin, whereas HG exposure played an opposite effect and reversed the regulatory effect of VEGFA silence on these markers (Fig. 2D). Furthermore, the FITC-dextran assay was also used to evaluate cell permeability. As shown in Fig. 2E, VEGFA interference significantly inhibited the flux of FITC-dextran in ARPE-19 cells, but HG treatment increased the flux of FITC-dextran and abrogated the suppressive role of VEGFA silence. In addition, EMT- and permeability-associated markers were measured by western blotting in ARPE-19 cells. VEGFA knockdown resulted in evident upregulation of Occludin and E-cadherin, and downregulation of N-cadherin, Vimentin, and Snail1 (Fig. 2F). However, HG treatment induced an opposite effect and it reversed the regulatory function of VEGFA silence on these proteins (Fig. 2F). These data indicated VEGFA interference constrained HG-induced EMT and cell permeability of ARPE-19 cells.

### YY1 knockdown downregulates VEGFA and Snail1 expression, and inhibits HG-induced EMT and cell permeability of ARPE-19 cells

**Fig. 3:**
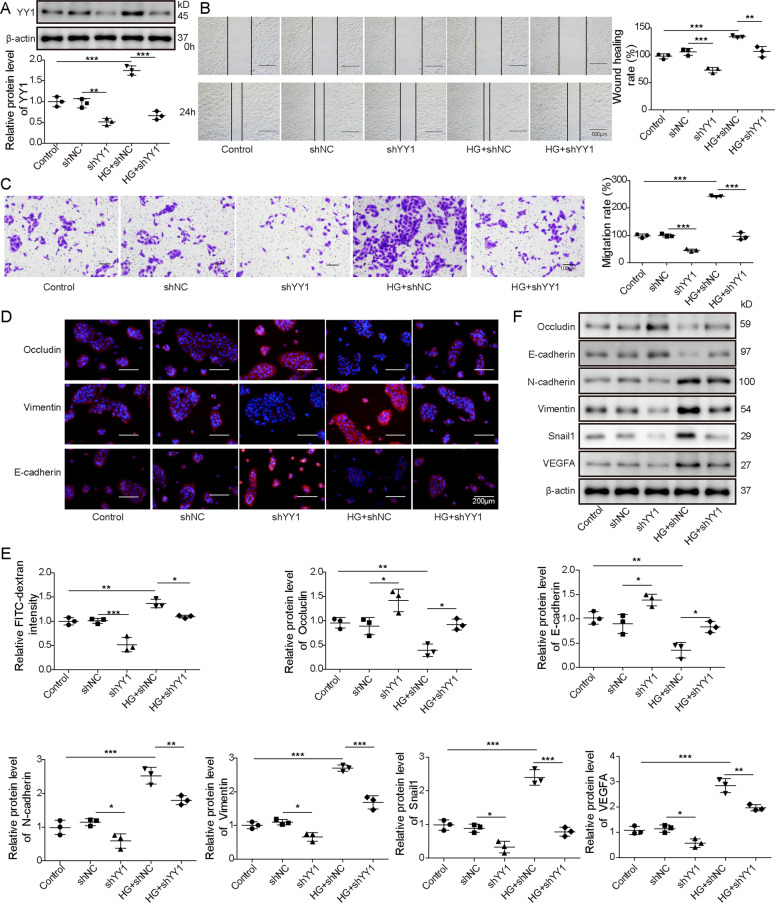
The inhibitive effect of YY1 knockdown on expression of VEGFA and Snail1, and HG-induced EMT and cell permeability of ARPE-19 cells. ARPE-19 cells were transfected with shNC or shYY1 before treatment of HG. **A** YY1 level was detected by western blotting in the treated cells. **B**, **C** The migratory ability was examined via wound-healing analysis and transwell assay in the treated cells. **D** Occludin, E-cadherin, and Vimentin abundances were detected via immunofluorescence in the treated cells. **E** Cell permeability was analyzed by FITC-dextran assay in the treated cells. **F** VEGFA, Snail1, Occludin, E-cadherin, N-cadherin, and Vimentin abundances were measured by western blotting in the treated cells. For each analysis, three technical replicates were performed and three biological independently performed replicates are included, *p < 0.05, **p < 0.01, ***p < 0.001.

To analyze the role of YY1 in HG-stimulated ARPE-19 cells, ARPE-19 cells were transfected with shNC or shYY1 prior to HG stimulation. The transfection of shYY1 significantly reduced YY1 abundance in ARPE-19 cells, which was restored via HG stimulation (Fig. 3A). Furthermore, as shown by wound-healing and transwell assays, YY1 silence markedly inhibited migration of ARPE-19 cells and this effect was abolished by HG stimulation (Fig. 3B, C). In addition, YY1 knockdown evidently suppressed the permeability of ARPE-19 cells by increasing expression of Occludin and E-cadherin, and decreasing Vimentin and FITC-dextran flux levels, and these events were reversed via HG stimulation (Fig. 3D, E). Moreover, YY1 interference significantly enhanced expression of Occludin and E-cadherin, and reduced abundances of N-cadherin, Vimentin, Snail1, and VEGFA in ARPE-19 cells, whereas HG stimulation reversed the regulatory effect of YY1 silence on these proteins (Fig. 3F). These results suggested YY1 interference repressed HG-induced EMT and cell permeability of ARPE-19 cells.

### YY1 regulates VEGFA and Snail1 expression, and is degraded via Smurf2 through ubiquitination

**Fig. 4:**
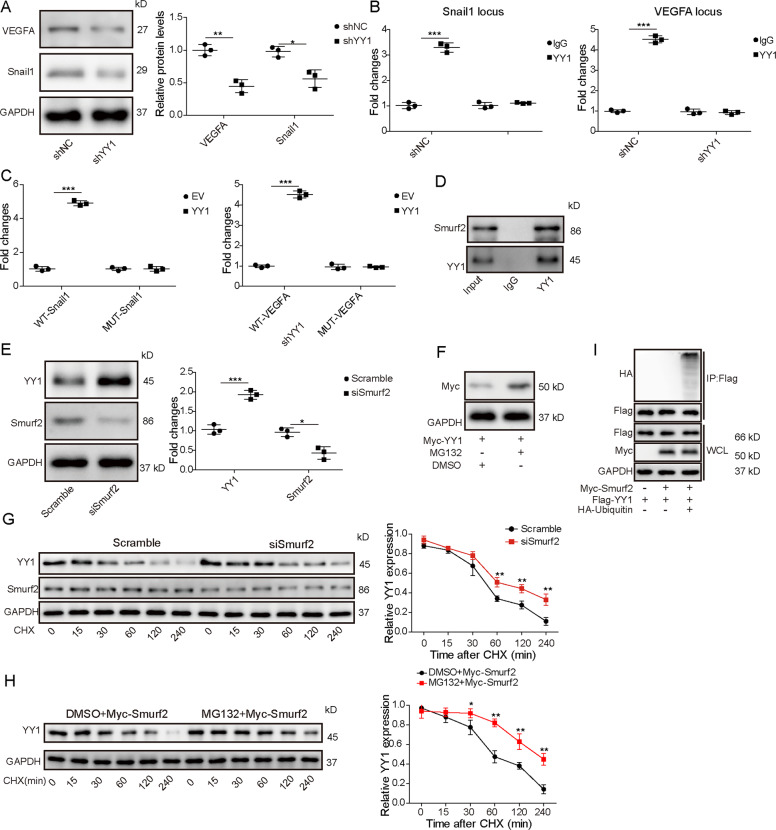
YY1 binds with VEGFA and Snail1, and ubiquitination and degradation of YY1 are mediated by Smurf2. **A** VEGFA and Snail1 levels were detected by western blotting in ARPE-19 cells transfected with shNC or shYY1. **B** The binding of YY1 on the promoter of VEGFA and Snail1 was analyzed by ChIP in ARPE-19 cells transfected with shNC or shYY1. **C** Luciferase activity was measured in ARPE-19 cells co-transfected with empty vector (EV) or YY1 overexpression vector and WT-Snail1, Mut-Snail1, WT-VEGFA, or Mut-VEGFA. **D** Smurf2 and YY1 levels were detected by western blotting after Co-IP of YY1 antibody. **E** YY1 and Smurf2 levels were measured via western blotting in ARPE-19 cells transfected with siSmurf2 or Scramble. **F** Western blotting analysis of YY1 levels in WCL derived from HEK293T cells with or without MG132 treatment. **G** YY1 and Smurf2 levels were examined by western blotting in ARPE-19 cells transfected with siSmurf2 or Scramble before treatment of cycloheximide (CHX) for different time points. **H** Smurf2-overexpressed HEK293T cells were treated with CHX for 0, 15, 30, 60, 120, and 240 min in the presence of MG132 (a proteasome inhibitor), followed by the detection of YY1 using western blotting. **I** Expression vectors encoding Flag-YY1 and HA-ubiquitin were co-transfected into HEK293T cells transfecting Myc-Smurf2 and cell lysates were subjected for Co-IP using anti-Flag, which was followed by western blotting using anti-HA antibody. For each analysis, three technical replicates were performed and three biological independently performed replicates are included, *p < 0.05, **p < 0.01, ***p < 0.001.

To explore how YY1 could regulate VEGFA and Snail1, the effect of YY1 silence on their expression was investigated in ARPE-19 cells. YY1 knockdown using shYY1 obviously decreased VEGFA and Snail1 protein levels (Fig. 4A). Moreover, the JASPAR algorithm predicted there were binding sites of YY1 on the promoter of VEGFA and Snail1. Further, ChIP assay showed that YY1 could bind with the promoter of VEGFA and Snail1 to initiate their expression (Fig. 4B). In addition, the WT-Snail1, Mut-Snail1, WT-VEGFA, and Mut-VEGFA luciferase reporter vectors were constructed. The dual-luciferase reporter assay showed YY1 overexpression increased the luciferase activity of WT-Snail1 or WT-VEGFA, but it did not affect the activity of Mut-Snail1 or Mut-VEGFA (Fig. 4C). In addition, we also predicted that there were three binding sites (BS1, BS2, and BS3) of YY1 on the promoter of Vimentin using JASPAR algorithm (Fig. S2A). Then, ChIP assay showed that YY1 could bind with the BS2 promoter of Vimentin to initiate its expression (Fig. S2B). In addition, the dual-luciferase reporter assay showed YY1 can directly interact with Vimentin (Fig. S2C). These results suggested that YY1 could bind with Vimentin, VEGFA, and Snail1. Furthermore, the interaction of YY1 and Smurf2 was analyzed in ARPE-19 cells. The Co-IP analysis showed that YY1 could bind with Smurf2 (Fig. 4D). Moreover, YY1 protein expression was evidently increased via Smurf2 knockdown using siSmurf2 (Fig. 4E). The half-life of YY1 was markedly prolonged after MG132 (a protein synthesis inhibitor) treatment (Fig. 4F). In addition, to analyze whether YY1 was degraded by Smurf2-mediated ubiquitination, ARPE-19 cells were transfected with Scramble or siSmurf2 before exposure to 100 μg/mL of cycloheximide (CHX) for different times. As shown in Fig. 4G, the degradation of YY1 was lowered by Smurf2 knockdown in the presence of CHX. To further confirm whether Smurf2 mediated the YY1 ubiquitination, Smurf2-overexpressed HEK293T cells were treated with CHX for 0, 15, 30, 60, 120, and 240 min in the presence of MG132 (a proteasome inhibitor), followed by the detection of YY1 using western blotting. As results indicated, Smurf2 significantly reduced the half-life of YY1 in the presence of CHX, whereas this phenomenon was blocked by MG132 treatment (Fig. 4H). Moreover, expression vectors encoding Flag-YY1 and HA-ubiquitin were co-transfected into HEK293T cells transfecting Myc-Smurf2, and cell lysates were subjected for Co-IP using anti-Flag, which was followed by western blotting using anti-HA antibody. As results suggested, YY1 ubiquitination was only observed in the presence of HA-ubiquitin and Myc-Smurf2 (Fig. 4I), indicating that Smurf2 mediated YY1 ubiquitination. These findings showed YY1 could modulate VEGFA and Snail1 expression, and was degraded via Smurf2-mediated ubiquitination.

### MiR-195 knockdown regulates Smurf2 and YY1 levels, and suppresses HG-induced EMT and cell permeability of ARPE-19 cells

**Fig. 5:**
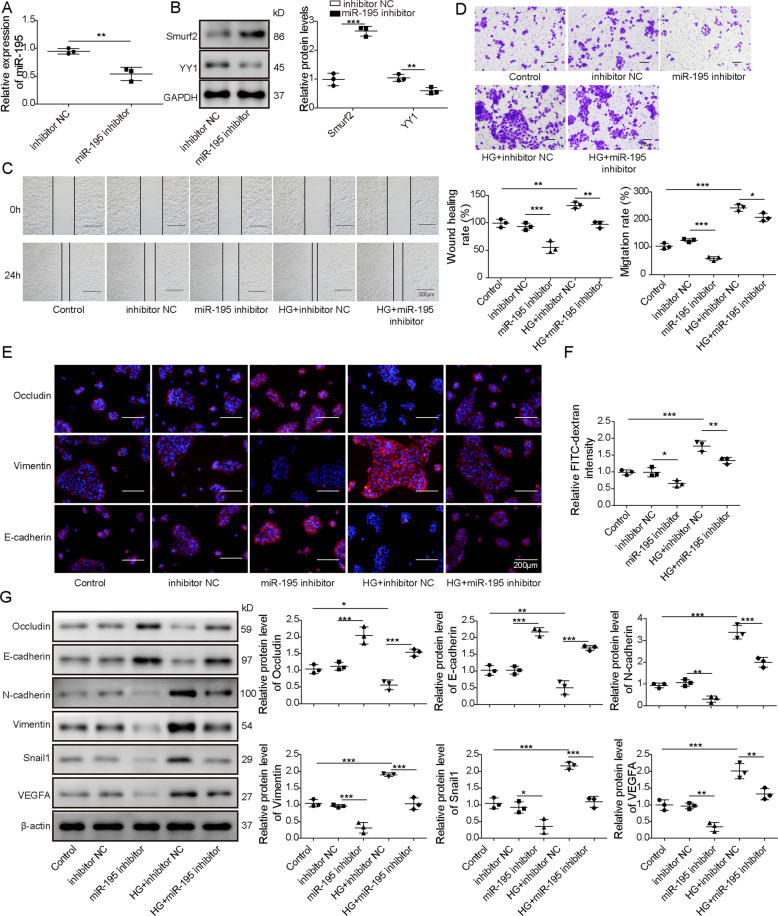
MiR-195 knockdown upregulates Smurf2 to inhibit YY1 and represses HG-induced EMT and cell permeability of ARPE-19 cells. ARPE-19 cells were transfected with inhibitor NC or miR-195 inhibitor, and were then stimulated or not stimulated by HG. **A** MiR-195 abundances were measured via qRT-PCR in the transfected cells. **B** Smurf2 and YY1 abundances were measured via western blotting in the transfected cells. **C**, **D** Cell migration was measured using wound-healing analysis and transwell assay in the treated cells. **E** Occludin, E-cadherin, and Vimentin abundances were determined by immunofluorescence in the treated cells. **F** Cell permeability was evaluated via FITC-dextran assay in the treated cells. **G** VEGFA, Snail1, Occludin, E-cadherin, N-cadherin, and Vimentin levels were detected via western blotting in the treated cells. For each analysis, three technical replicates were performed and three biologically independently performed replicates are included, *p < 0.05, **p < 0.01, ***p < 0.001.

To explore the role of miR-195 in HG-stimulated ARPE-19 cells, ARPE-19 cells were transfected with inhibitor NC or miR-195 inhibitor before HG stimulation. The transfection of miR-195 inhibitor effectively reduced miR-195 abundance in ARPE-19 cells and miR-195 knockdown significantly enhanced Smurf2 expression and decreased YY1 level (Fig. 5A, B). Moreover, miR-195 knockdown clearly restrained ARPE-19 cell migration, which was reversed via HG stimulation (Fig. 5C, D). Furthermore, miR-195 downregulation significantly repressed the permeability of ARPE-19 cells via increasing abundances of Occludin and E-cadherin, and reducing Vimentin and FITC-dextran flux levels, and this effect was abolished via HG stimulation (Fig. 5E, F). In addition, miR-195 knockdown evidently increased levels of Occludin and E-cadherin, and decreased abundances of N-cadherin, Vimentin, Snail1, and VEGFA in ARPE-19 cells, but HG stimulation relieved the regulatory role of miR-195 silence (Fig. 5G). These data indicated that miR-195 knockdown inhibited HG-induced EMT and cell permeability of ARPE-19 cells.

### MiR-195 targets Smurf2 to regulate expression of YY1, VEGFA, and Snail1, and HG-induced EMT and cell permeability of ARPE-19 cells

**Fig. 6:**
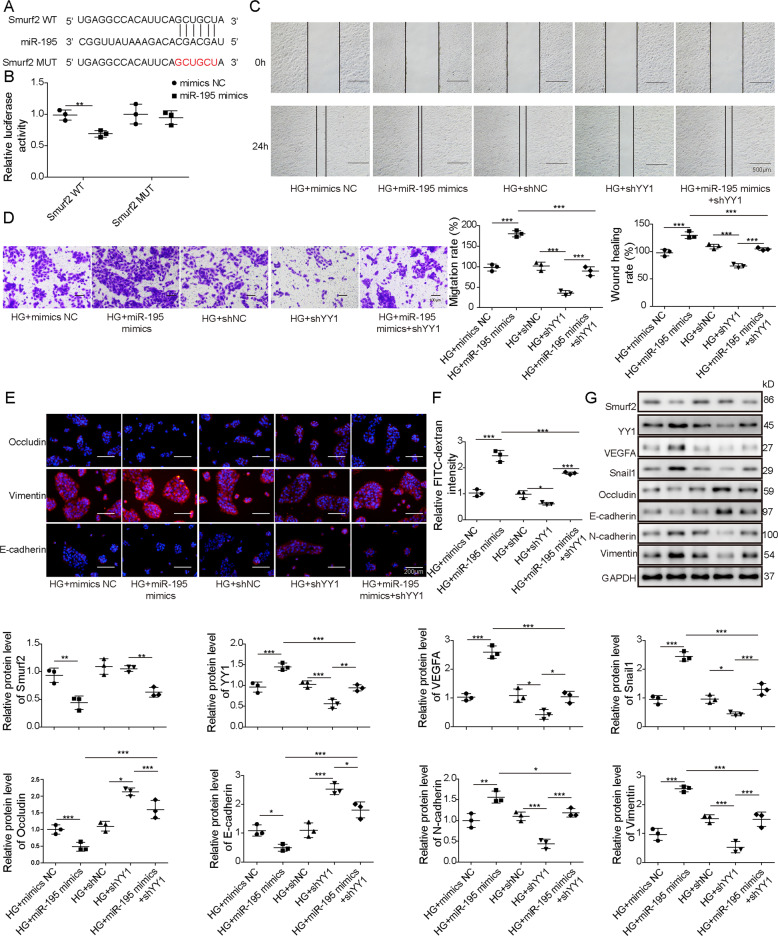
MiR-195 targets Smurf2 to upregulate expression of YY1, VEGFA, and Snail1, and promote HG-induced EMT and cell permeability of ARPE-19 cells. **A** The target sites of miR-195 on Smurf2 were predicted via StarBase. **B** Luciferase activity was detected in ARPE-19 cells co-transfected with mimics NC or miR-195 mimics and Smurf2 WT or Smurf2 Mut. ARPE-19 cells were transfected with mimics NC, miR-195 mimics, shNC, shYY1, or miR-195 mimics + shYY1 before treatment of HG. **C**, **D** Cell migration was detected via wound-healing analysis and transwell assay in the treated cells. **E** Occludin, E-cadherin, and Vimentin levels were detected via immunofluorescence in the treated cells. **F** Cell permeability was investigated using FITC-dextran analysis in the treated cells. **G** Smurf2, YY1, VEGFA, Snail1, Occludin, E-cadherin, N-cadherin, and Vimentin levels were measured by western blotting in the treated cells. For each analysis, three technical replicates were performed and three biologically independently performed replicates are included, *p < 0.05, **p < 0.01, ***p < 0.001.

To explore how miR-195 could regulate Smurf2 expression, the binding potential was predicted by StarBase. The target sites of miR-195 on Smurf2 were shown in Fig. 6A. Moreover, the Smurf2 WT and Smurf2 Mut luciferase reporter vectors were constructed. MiR-195 mimics obviously reduced the luciferase activity of Smurf2 WT, but it did not change the activity of Smurf2 Mut, which suggested miR-195 could target the 3′-UTR of Smurf2 (Fig. 6B). In addition, to explore whether YY1 was involved in miR-195-modulated cell processes, ARPE-19 cells were transfected with mimics NC, miR-195 mimics, shNC, shYY1, or miR-195 mimics + shYY1 before HG stimulation. The data of wound-healing assay showed miR-195 mimics significantly promoted cell migration in HG-stimulated ARPE-19 cells, and this effect was weakened via YY1 silence using shYY1 (Fig. 6C, D). Furthermore, miR-195 overexpression markedly facilitated the permeability of HG-stimulated ARPE-19 cells via decreasing the expression of Occludin and E-cadherin, and increasing Vimentin and FITC-dextran flux levels, and this influence was mitigated via YY1 knockdown (Fig. 6E, F). In addition, miR-195 overexpression markedly decreased levels of Smurf2, Occludin, and E-cadherin, and increased abundances of YY1, N-cadherin, Vimentin, Snail1, and VEGFA in HG-stimulated ARPE-19 cells, and this regulatory effect of miR-195 mimics on protein expression was reversed via YY1 interference (Fig. 6G). To further confirm the regulatory effects of miR-195 on HG-induced EMT and cell permeability of ARPE-19 cells were mediated by Smurf2, we conducted a series of rescue assays in ARPE-19 cells transfected with miR-195 mimics or/and Smurf2. The overexpression efficiency of Smurf2 under normal and HG condition was verified by western blotting (Fig. S1A). Using wound-healing and transwell assays, we revealed that the promotive effects of miR-195 mimics on cell migration of ARPE-19 cells under HG condition were blocked by Smurf2 overexpression (Fig. S1B, C). Also, the promotive impacts of miR-195 mimics on cell permeability of HG-stimulated ARPE-19 cells were abrogated by Smurf2 overexpression (Fig. S1D, E). Moreover, miR-195 overexpression markedly decreased levels of Smurf2, Occludin, and E-cadherin, and increased abundances of YY1, N-cadherin, Vimentin, Snail1, and VEGFA in HG-stimulated ARPE-19 cells, and this regulatory effect of miR-195 mimics on protein expression was abrogated by via Smurf2 overexpression (Fig. S1F). These findings suggested miR-195 could promote HG-induced EMT and cell permeability of ARPE-19 cells under HG condition by targeting Smurf2.

## Discussion

Diabetic retinopathy is a specific complication of diabetes and new therapies such as anti-VEGF treatments have gained breakthrough, while the cost-effectiveness remains a big issue [23]. Hence, it is of importance to explore innovative strategies for diabetic retinopathy treatment. MiRNA-targeted invention might open new opportunity for the therapy of diabetic microvascular diseases, including diabetic retinopathy [24]. In this study, we explored and confirmed miR-195 might promote YY1-initiated VEGFA/Snail1 expression by inhibiting Smurf2-mediated ubiquitination, further increasing EMT and permeability of HG-treated ARPE-19 cells.

STZ-induced diabetic model was widely used for the studies on diabetic retinopathy [25, 26]. Here we established this murine model and found retinal structure change in the model group. Moreover, we found the dysregulated miR-195, YY1, VEGFA, and Snail1 were associated with diabetic retinopathy development. To study their functions in diabetic retinopathy, we further established the cellular model using HG-stimulated ARPE-19 cells as previous reports [10, 27]. We confirmed HG induced EMT and permeability of ARPE-19 cells, which indicated the successful establishment of diabetic retinopathy model. Snail1 acted as a mesenchymal marker, which contributed to EMT process in diabetic retinopathy [7, 28]. Moreover, Snail1 could promote cell permeability in intestinal barrier dysfunction and glomerular endothelium damage [29, 30]. VEGFA could drive Snail1 nuclear localization to regulate Snail1-mediated EMT [11, 12, 31]. In addition, VEGFA facilitated retinal vessel permeability in diabetic retinopathy [32, 33]. These all suggested Snail1 and VEGFA could promote EMT and cell permeability. Similarly, our study validated VEGFA could upregulate Snail1 to increase EMT and cell permeability of HG-stimulated ARPE-19 cells, which was also consistent with the findings in a previous study [10].

Then we explored how VEGFA and Snail1 were initiated in diabetic retinopathy. YY1 could promote EMT and fibrosis in diabetic nephropathy [34]. Moreover, YY1 could increase vascular endothelial permeability in cholangiocarcinoma [35]. Consistent with these reports, we also confirmed YY1 promoted EMT and cell permeability of HG-stimulated ARPE-19 cells. Previous reports indicated YY1 could initiate VEGFA and Snail1 via interacting with their promoter [14, 15]. Similarly, here we also found YY1 could upregulate VEGFA and Snail1 expression to regulate diabetic retinopathy development. Furthermore, multiple evidences have reported Smurf2 could degrade YY1 expression by ubiquitination [21, 36, 37]. Then, the Smurf2/YY1/Snail1 axis is an important network for EMT process [38]. Here we first confirmed Smurf2 could inhibit YY1 level by ubiquitination to regulate VEGFA/Snail1-derived EMT and cell permeability of HG-stimulated ARPE-19 cells.

Next, we explored the upstream of Smurf2 and validated miR-195 could target Smurf2 in ARPE-19 cells, which was also in agreement with the finding in lung cancer cells [22]. MiR-195 was upregulated in retinal tissues of STZ-induced diabetic rats and promoted the oxidative stress and inflammatory injury [39]. Moreover, miR-195 facilitated the oxidative stress, mitochondrial damage, and apoptosis of HG-treated ARPE-19 cells [40]. In addition, miR-195 increased the tube formation and permeability of diabetic retinal vasculature by targeting mitofusin 2 in diabetic rats [19]. These reports suggested miR-195 might induce worse function in diabetic nephropathy. Our study first found miR-195 contributed to EMT and cell permeability of HG-stimulated ARPE-19 cells by targeting Smurf2/YY1 axis. However, there was a limitation in our study. We did not investigate the function of miR-195/Smurf2/YY1/VEGFA/Snail1 axis in clinical or preclinical experiments, which would be explored in the future.

**Fig. 7:**
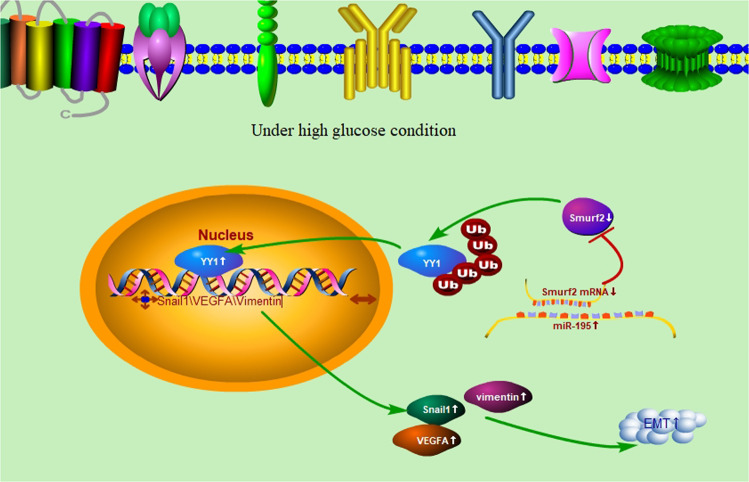
Proposed model of the role of miR-195 for EMT and permeability in HG-stimulated ARPE-19 cells. miR-195 increased EMT and cell permeability mediated via VEGFA/Snail1 axis in HG-stimulated ARPE-19 cells, possibly via upregulating YY1 indirectly by targeting Smurf2.

In conclusion, miR-195 increased EMT and cell permeability mediated via VEGFA/Snail1 axis in HG-stimulated ARPE-19 cells, possibly via upregulating YY1 indirectly by targeting Smurf2. This study provided a new mechanism for understand the EMT and cell permeability of retinal pigment epithelial cells, and indicated novel target for diabetic nephropathy treatment (Fig. 7).

## Supplementary information

Supplemental Figure Legends

SUPPLEMENTAL Figure 1

SUPPLEMENTAL Figure 2
