# Supplementary material for: MiR-195 inhibits the ubiquitination and degradation of YY1 by Smurf2, and induces EMT and cell permeability of retinal pigment epithelial cells
Source: Cell Death Dis. 2021 Jul 15;12(7):708. doi: 10.1038/s41419-021-03956-6 (PMC8282777; doi:10.1038/s41419-021-03956-6)
Supplement: Supplementary file 1 — Supplemental Figure Legends [file 41419_2021_3956_MOESM1_ESM.docx]

**Supplemental Figures**

**Supplemental Figure 1. Overexpression of Smurf2 abolished the effects of miR-195 on regulation of YY1 and HG-induced EMT and cell permeability of ARPE-19 cells.** (A) Western blot was used to detect the expression of Smurf2 in ARPE-19 cells transfected with Smurf2 or NC under normal or HG environment. (B and C) Cell migration of ARPE-19 cells transfected with miR-195 mimics or/and Smurf2-overexpression vector was detected via wound healing and transwell assays. (D) Occludin, E-cadherin and Vimentin levels were examined by immunofluorescence in ARPE-19 cells each group. (E) Cell permeability was investigated using FITC-dextran analysis in each group. (F) The protein expression of Smurf2, YY1, VEGFA, Snail1, Occludin, E-cadherin, N-cadherin and Vimentin in miR-195 mimics or/and Smurf2-overexpression vector treated ARPE-19 cells were examined by western blot. For each analysis, three technical replicates were performed and three biologically independently performed replicates are included, **p* < 0.05, ***p* < 0.01, ****p* < 0.001.

**Supplemental Figure 2.** (A) The JASPAR algorithm predicted there are three binding sites (BS1, BS2, and BS3) of YY1 on the promoter of Vimentin. (B) The binding of YY1 on the promoter of Vimentin was analyzed by ChIP in ARPE-19 cells treated with YY1 or IgG. (C) Luciferase activity was measured in ARPE-19 cells co-transfected with EV or YY1 overexpression vector and BS1-mut, BS2-mut, and BS3-mut. For each analysis, three technical replicates were performed and three biologically independently performed replicates are included, ****p* < 0.001.
