# Supplementary figures and images for: MiR-195 inhibits the ubiquitination and degradation of YY1 by Smurf2, and induces EMT and cell permeability of retinal pigment epithelial cells
Source: Cell Death Dis. 2021 Jul 15;12(7):708. doi: 10.1038/s41419-021-03956-6 (PMC8282777; doi:10.1038/s41419-021-03956-6)

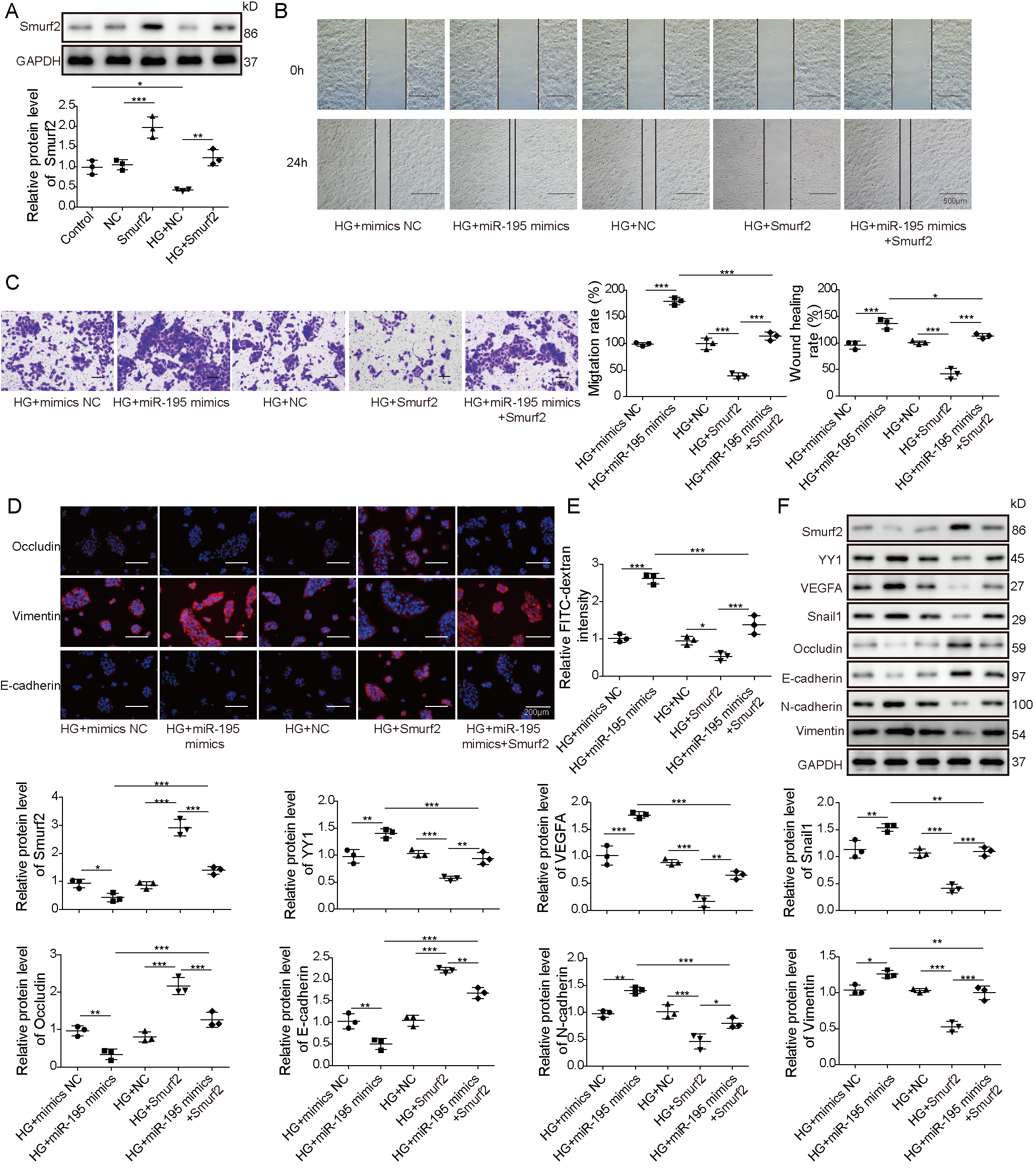

Supplement: Supplementary file 2 — SUPPLEMENTAL Figure 1 [file 41419_2021_3956_MOESM2_ESM.tif]

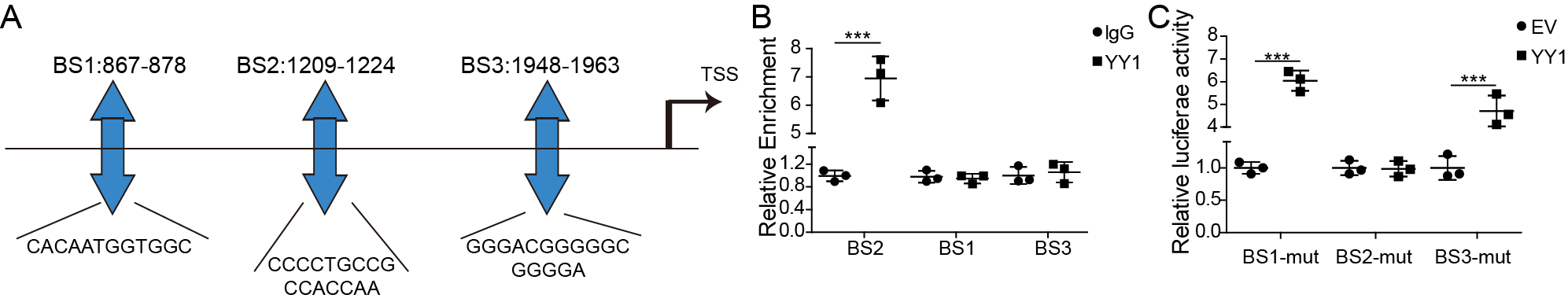

Supplement: Supplementary file 3 — SUPPLEMENTAL Figure 2 [file 41419_2021_3956_MOESM3_ESM.tif]
